# Etiology of childhood diarrhoea among under five children and molecular analysis of antibiotic resistance in isolated enteric bacterial pathogens from a tertiary care hospital, Eastern Odisha, India

**DOI:** 10.1186/s12879-019-4501-6

**Published:** 2019-12-02

**Authors:** Sonam S. Moharana, Rakesh K. Panda, Muktikesh Dash, Nirupama Chayani, Priyanka Bokade, Sanghamitra Pati, Debdutta Bhattacharya

**Affiliations:** 1 Dept. of Microbiology, SCB Medical College (Govt. of Odisha), Manglabag, Cuttack, Odisha 753007 India; 2 Dept. of Bacteriology & One Health, ICMR-Regional Medical Research Centre (Dept. of Health Reserch, Ministry of Health & Family Welfare, Govt. of India), Chandrasekharpur, Bhubaneswar, 751023 India

**Keywords:** Childhood diarrhoea, Under 5 years, Etiology, *E.coli*, Multidrug-resistant, Mutation, QRDR, DEC, Surveillance

## Abstract

**Background:**

Although, India has made steady progress in reducing deaths in children younger than 5 years, the proportional mortality accounted by diarrhoeal diseases still remains high. The present hospital based cross sectional study was carried out to understand the prevalence of various bacterial pathogens associated with the diarrhoea cases in under 5 years age group.

**Methods:**

During, 1st September, 2015 to 30th November 2017, all the childhood diarrhoea cases (≤5 yrs) of SCB Medical College in Odisha, India were included in the study. Stool samples were collected and processed for the isolation of causative bacterial pathogen and the isolated bacterial pathogens were subjected to antibiotic sensitivity testing, molecular analysis of drug resistance. Clinical and demographic data were collected and analyzed.

**Results:**

Three hundred twenty patients were enrolled in the study during the study period from whom 82 bacterial isolates were obtained indicating a proportional causality of 25.6% for bacterial diarrhoea among children in this region. Entero toxigenic *E.coli* (ETEC) accounted for majority of the cases and and more than 50% of the strains were found to be multi-drug resistant (resistant to more than 3 class of antibiotics). More than 50% of the strains were resistant to current choice of treatment like ciprofloxacin, ofloxacin and ceftriaxone and 2.4% being resistant to Imipenem. ESBL production was also observed in some of the strains and one isolate harboured the *NDM-1* gene. Fluoroquinolone resistance was found to be linked with multiple mutations in the QRDR region followed by PMQR determinants.

**Conclusion:**

The current study, to the best of our knowledge is first of its kind which demonstrated the etiology of bacterial diarrhoea in children less than 5 years old and identified diarrheogenic *E. coli* as the predominant enteropathogen in Odisha. Majority of the isolates being multi-drug resistance calls for a continuous surveillance system in the region which will be helpfulin identifying emerging resistance pattern and for developing suitable intervention stategies.

## Background

In developing countries, diarrhoea continues to be one of the major contributing factor for morbidity and mortality among children and ranks 2nd most common cause of death among the group under 5 years of age globally after respiratory illness [1]. Rapid progress has been made by India in reducing deaths in children under 5 years, with total deaths declining from 2.5 to 1.5 million during 2001–2012 [2] which was due to various programs adopted by the government on Immunization and Integrated Management of Childhood illnesses (IMNCI) [3]. However, the proportional mortality due to acute diarrhoeal diseases still remains high.

An estimated 300,000 children die each year in India making diarrhoea as the 3rd major cause of death in children below 5 years of age (MDSC, 2010). National Institute of Cholera and Enteric Disease, Kolkata, (India), reported crude death rate due to diarrhoea in rural India as 9.3 per 1000 populations and 22% of the total deaths among 0–6 year age due to diarrhoeal disease [4]. The highest percentage of children suffering from diarrhoea belonged to states of Madhya Pradesh, Odisha and Tamil Nadu [5].

Among developing countries across the world, *Rotavirus* and *Escherichia coli* are the two most common causes of diarrhoea, whereas *Campylobacter* spp. is significantly responsible for diarrhoea in developed countries [6]. The most common diarrhoeagenic pathogen include *Escherichia coli, Rotavirus, Salmonella* spp*., Shigella* spp*., Entamoeba histolytica and* enterotoxigenic *Bacteroides fragilis, Campylobacter jejuni, Cryptosporidium spp* [7, 8]*.*

Antibiotic resistance has emerged as a major public health threat in India. A high burden of infectious diseases, unregulated sale of antibiotics, financial incentives for healthcare providers to prescribe antibiotics, patient expectations, rising incomes, and limited public health response have helped drive the emergence of resistance [9, 10]. Rapid emergence of resistance among these common pathogens is posing a serious threat.

The present study is a hospital based cross sectional study to understand the prevalence of various bacterial pathogens associated with the childhood diarrhoea cases admitted/attending largest government tertiary care hospital in Odisha, India. The result obtained from this study will be helpful in identifying the prevalent bacterial pathogens associated with acute diarrhoeal disease (ADD) in children under 5 years, emerging antimicrobial resistance trends and formulate interventional strategies for effective management and control of ADD in under five children.

## Methods

### Patients and samples

Childhood diarrhoea cases (≤5 yrs) who were either attending or admitted the SCB Medical College (largest government-run medical college and hospital in Odisha) during 1st September, 2015 to 30th November 2017 were included in the study. After obtaining signed consent, stool samples were collected and processed for the isolation and identification of causative agent.

### Microbiological examination

The stool samples were subjected to standard techniques for isolation and identification of the bacterial isolates using suitable growth media [11]. After overnight incubation at 37 °C, a single colony was isolated from the selective media and grown in pure culture and subsequently identified using standard procedure in which the suspected colonies were subjected to biochemical and serological tests for *Vibrio* sp., *Shigella* sp. and *Salmonella enterica sp. using* group-specific antisera (Denka Seiken Co., Ltd., Tokyo, Japan). Wherever more than a single type of colony was observed, all the suspected colonies were subjected to isolation and identification. The *E. coli* isolated on culture plate from a diarrhoea stool were subjected to PCR based identification and pathotyping.

### Antimicrobial resistance profiling

Antibiotic sensitivity tests were performed by a disk diffusion method for 11 drugs (Amikacin AMK; Cotrimoxazole, CoT; Imipenem, IPM; Tetracycline, TET; Nalidixic acid, NAL; Ofloxacin, OFX; Ciprofloxacin, CIP; Levofloxacin, LEV; Ceftriaxone, CRO; Ampicillin, AMP; Amoxicillin/clavulanic acid, AMC) following the Clinical and Laboratory Standards Institute (CLSI) guidelines, 2015 [12]. Control strains of *Escherichia coli* ATCC 25922 and *Staphylococcus aureus* ATCC 25923 were included in each test. Multi-drug resistance was defined as resistant to 3 or more antimicrobial categories [13].

### ESBL production

The isolates exhibiting resistance to to third-generation cephalosporins were tested for the production of extended spectrum β-lactamase (ESBL) using the combination disk test with ceftazidime–clavulanic acid (CAC, 30/10 μg) and ceftriaxone–clavulanic acid [11, 13].

### Minimum inhibitory concentrations

Etest (AB Biodisk, Solna, Sweden) was used for detection of minimum inhibitory concentrations (MICs) for quinolones (NAL) and fluoroquinolones (CIP and OFX), which was interpreted as per the CLSI guidelines [11].

### Detection of diarrhoeagenic E.coli by multiplex PCR

DNA was isolated following the heat–chill method [14]. The template DNA was used in PCR for detection of various DEC like Enterotoxigenic *E.coli* (ETEC), Enteropathogenic *E.coli* (EPEC), Enteroaggregative *E.coli* (EAEC) and Enterohemorrhagic *E.coli* (EHEC) [15]. The isolates were screened for various virulent genes specific for ETEC (*elt, est*), EPEC (*bfpA, eae*), EAEC (*aatA, aaiC*) and EHEC (*stx1, stx2*) using standard procedure (Table 1). The strains harboring these particular genes were considered positive for the respective strains of DEC. DNA from previously confirmed strains belonging to the 4 types of DEC were included in each PCR assay as positive control. Sterile double distilled water was used as negative control.

**Table 1:** PCR primers used in the study

Gene	Sequence (5′-3′)	Annealing temp (°C)	Amplicon size (bp)
*elt*	CACACGGAGCTCCTCAGTC	57	508
	CCCCCAGCCTAGCTTAGTTT		
*est*	GCTAAACCAGTAG/AGGTCTTCAAAA	57	147
	CCCGGTACAG/AGCAGGATTACAACA		
*bfpA*	GGAAGTCAAATTCATGGGGG	57	367
	GGAATCAGACGCAGACTGGT		
*AatA*	CTGGCGAAAGACTGTATCAT	57	630
	CAATGTATAGAAATCCGCTGTT		
*eae*	CCCGAATTCGGCACAAGCATAAGC	57	881
	CCCGGATCCGTCTCGCCAGTATTCG		
*aaiC*	ATTGTCCTCAGGCATTTCAC	57	215
	ACGACACCCCTGATAAACAA		
*Stx1*	CAACACTGGATGATCTCAG	57	350
	CCCCCTCAACTGCTAATA		
*Stx2*	ATCAGTCGTCACTCACTGGT	57	110
	CTGCTGTCACAGTGACAAA		
*bla*_*CTX-M3*_	AATCACTGCGTCAGTTCAC	50	701
	TTTATCCCCCACAACCCAG		
*NDM-1*	ACCGCCTGGACCGATGACCA	58	264
	GCCAAAGTTGGGCGCGGTTG		
*gyrA*	TACACCGGTCAACATTGAGG	64	648
	TTAATGATTGCCGCCGTCGG		
*gyrB*	TGAAATGACCCGCCGTAAAGG	64	309
	GCTGTGATAACGCAGTTTGTCCGGG		
*parC*	GTCTGAACTGGGCCTGAATGC	64	249
	AGCAGCTCGGAATATTTCGACAA		
*parE*	ATGCGTGCGGCTAAAAAAGTG	64	290
	TCGTCGCTGTCAGGATCGATAC		
*qnrA*	ATTTCTCACGCCAGGATTTG	64	516
	GATCGGCAAAGGTTAGGTCA		
*qnrB*	GATCGTGAAAGCCAGAAAGG	64	476
	ATGAGCAACGATGCCTGGTA		
*qnrC*	GGGTTGTACATTTATTGAATCG	64	307
	CACCTACCCATTTATTTTCA		
*qnrS*	GCAAGTTCATTGAACAGGGT	64	428
	TCTAAACCGTCGAGTTCGGCG		
*aac(6′)-Ib-cr*	TTGCGATGCTCTATGAGTGGCTA	55	482
	CTCGAATGCCTGGCGTGTTT		
*qepA*	AACTGCTTGAGCCCGTAGAT	55	596
	GTCTACGCCATGGACCTCAC		

### Mutation analysis in QRDRs

The quinolone resistance determining regions (QRDRs) of the *gyrA, gyrB, parC*, and *parE* genes were amplified using published primers (Table 1), as reported previously [16]. Positive and negative control was used to establish the validity of the PCR assay. PCR products were then sequenced in an automatic sequencer (ABI 3130; Applied Biosystems, Foster City, CA, USA) followed by detection of mutation using SeqScape v3 (ABI 3130; Applied Biosystems).

### Screening of the PMQR determinants

The plasmid mediated quinolone resistance (PMQR) determinants which includes qnr determinants *qnrA, qnrB, qnrC, qnrS*, and two additional genes, *aac(6′)-Ib-cr* and *qepA* were screened for quinolone resistant strains using published primers (Table 1) [17, 18].

### Screening for the presence of NDM-1 gene

PCR based detection of *NDM-1* was performed for all strains resistant to Imipenem using published primers [19]. DNA isolated from previously isolated confirmed *NDM-1* strain of *P.putida* was used as positive control and sterile distilled water was used as negative control to establish the validity of the PCR assay.

### Statistical data analysis

Data was analysed statistically by SPSS version 21 software. Test used was Pearson chi square and chi square with Yates correction. *p* value < 0.05 was considered significant.

## Results

### Patients and isolates

**Fig. 1:**
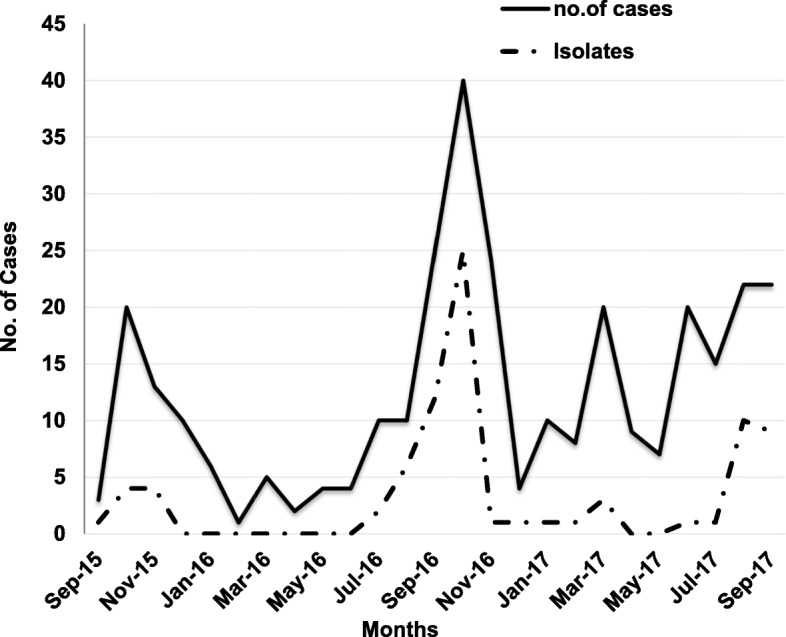
Seasonal pattern of distribution of acute diarrhoeal cases and pathogenic isolates

During the study, 320 patients were enrolled and sample were collected and processed from them, which includes 157 (49.06%) male and 163 (50.94%) female (Table 2). Bacteriological examination, revealed, 82 bacterial isolates from these samples, giving a proportional causality for bacterial diarrhoea of 25.6% among children in this region. No coinfection was observed. Each bacterial isolate was obtained from different individual cases i.e., 82 isolates were obtained from 82 different individual cases. The number of cases as well as isolation of bacterial pathogens were high during Sept-Oct during the 2 years period (Fig. 1). No deaths due to bacterial diarrhoea were reported during the study period. Of these 82 bacterial isolates, 77 (24%) were DEC, 2 (0.6%) were *V. cholerae*, 2 (0.6%) were *S. flexneri*, and 1 (0.3%) were *Salmonella enterica Paratyphi B*. PCR based identification of DEC revealed, 40 (51.9%) as ETEC, 33 (42.8%) as EPEC and 4 (5.1%) as EAEC.

**Table 2:** Prevalence of DEC pathotypes among the age group

DEC	Total	Age (in months)					Overall 2 values^a^
		0–6	7–24	25–36	37–48	49–60	
ETEC-LT	13 (16.9)	0	1 (33.3)	1 (20.0)	9 (15.0)	2 (25.0)	20.312188
ETEC-ST	17 (22.1)	0	1 (33.3)	0	16 (26.7)	0	1.766234
ETEC-LT + ST	10 (12.9)	0	1 (33.3)	1 (20.0)	5 (8.3)	3 (37.5)	33.701461
aEPEC	19 (24.7)	0	0	2 (40.0)	16 (26.7)	1 (12.5)	10.480605
tEPEC	14 (18.2)	0	0	1 (20.0)	12 (20)	1 (12.5)	10.142045
EAEC	4 (5.2)	1 (100)	0	0	2 (3.3)	1 (12.5)	10.821834
Total	77	1	3	5	60	8	87.224367

^a^at 10% level of significance

### Pathotypes of DEC

Among the 77 DEC detected from 320 cases, 40 (51.9%) were identified as ETEC, 33 (42.8%) as EPEC and 4 (5.1%) as EAEC by PCR based detection of toxigenic genes (Table 2). ETEC with elt was higher than est. ETEC in the age group of 25–26 years and 49–60 years, however its was lower in the age group of 37–48 years. Prevalence of atypical EPEC (aEPEC) was found to be more than typical EPEC (tEPEC), however the difference was not statistically significant although overall prevalence of DEC pathotypes were statistically significant at 10%.

### Age-wise distribution

The patients were categorized into 5 age groups viz. 0–6, 7–24, 25–36, 37–48 and 49–60 months to understand the distribution of various pathogens among these age. The cases (30.6%; 98 of 320) as well as isolates (76.82%; 63 of 82) were found to be highest in the age group of 37–48 months (Table 3, Fig. 1).

**Table 3:** Basic information and clinical symptoms of the children with acute diarrhoea cases and isolation positive cases

Characteristics		Total cases (%) *N* = 320	Negative for bacterial isolation (%) *n* = 238	Positive for bacterial isolation (%) *n* = 82	*p* value
Age (in months)					
0–6		10 (3.1)	9 (3.8)	1(1.2)	0.250
7–24		78 (24.4)	75 (31.5)	3(3.7)	0.000
25–36		74 (23.1)	68 (28.5)	6(7.3)	0.000
37–48		98 (30.6)	35(14.7)	63(76.8)	0.000
49–60		60 (18.8)	51(21.4)	9(10.9)	0.036
Sex	Male	157 (49.06)	123 (51.6)	34 (41.5)	0.110
	Female	163 (50.94)	115(48.3)	48 (58.5)	0.110
Duration of diarrhoea	<3 days	61(19.1)	48 (20.1)	13 (15.9)	0.391
	3–6 days	176 (55.0)	108 (45.3)	68 (82.9)	0.000
	>6 days	1 (0.3)	0	1 (1.2)	–
Fever	37–39 °C	138(43.1)	107 (44.9)	31(37.8)	0.259
Vomiting		115(35.9)	91 (38.2)	24(29.3)	0.144
Dehydration		33(10.3)	16 (6.2)	17(20.7)	0.000
Anaemia		21(6.5)	13 (5.6)	8(9.6)	0.175
Abdominal Pain		127(39.6)	89 (37.3)	38(46.3)	0.153

### Clinical features

Mucous stool, abdominal pain, anaemia, vomiting, fever, and severe dehydration, were found to be associated with the patients enrolled (Table 3). Significant difference was not observed among these clinical presentations between total cases and isolation positive cases. Most of the DEC cases were associated with diarrhoea for more than 3 days. No significant difference was observed among the clinical features associated among the samples positive for bacterial isolation and negative except in case of duration of diarrhoea (3–6 days) and dehydration (Table 3).

### Antibiotic sensitivity among bacterial isolates

A wide range of antibiotic resistance was observed among the bacterial isolates with more than 50% of the isolates resistant to more than 50% of the drugs tested (Table 4). All the 82 (100%) isolates were multidrug-resistant. More than 60% of the DEC were resistant to CRO (3rd generation Cephalosporins) and Fluoroquinolones (OFX and CIP). Among the 49 strains resistant to CRO, only 3 isolates of EPEC showed the presence ESBL production of which all are aEPEC. All these 3 isolates harbored the *CTX-M3* gene. Among the 82 isolates, 2 (2.4%) DEC showed the resistance to Imipenem, one harbored carbapenemase gene *NDM-1*.

**Table 4:** Antibiotic resistance pattern of the bacterial isolates obtained during the study period

Organism	No of pathogenic organism (%) *n* = 82	AMK	CoT	IMP	TET	NAL	OFX	CRO	AMP	LEV	AMC	CIP
DEC	77 (94)	1	45 (61)	2	18 (28.1)	77 (100)	47 (61.1)	49 (63.7)	66 (86.6)	43 (55.9)	34 (44.2)	56 (74.4)
*S. flexneri*	2	0 (0)	0 (0)	0 (0)	1	2	1	1	2	2	2	2
*Salmonella Paratyphi B*	1	0 (0)	0 (0)	0 (0)	0 (0)	1	0 (0)	0 (0)	1	0 (0)	1	0 (0)
*V.cholerae O1*	2	0 (0)	2	0 (0)	0 (0)	2	0 (0)	0 (0)	1	1	1	1

### Fluoroquinolone resistance among the bacterial strains

All the bacterial isolates were resistant to nalidixic acid of which 11 were sensitive to the fluoroquinolones (FLQ). A total of 59 (71.9%) of strains were ciprofloxacin resistant and 48 (58.5%) were ofloxacin resistant respectively (Table 3). Minimum inhibitory concentration of the isolates ranged between 64 to > 256 μg/mL for nalidixic acid, 4 to > 256 μg/mL for ciprofloxacin and 16 to > 256 μg/mL for ofloxacin.

### Detection of mutation in QRDR region and screening of PMQR

Only 20 DEC isolates resistant to quinolones and fluoroquinolones were subjected to QRDR mutation and screening of PMQR, which include 5 NAL resistant and 15 FLQ resistant strains. Several mutations were detected in the QRDR region of the fluoroquinolone resistant *E.coli* isolates (Table 5).

**Table 5:** Mutations in the QRDR region of the DEC isolates

Sl No.	Strain No.		ABST for	MIC (μg/ml)			*gyrA*	*parC*	*qnrB*	*aac6’Ibcr*	
			Quinolone	NAL	CIP	OFX	S83-L	D87-N	S80-I		
1	DEC1	ETEC	NAL	128	–	–	+	–	–	–	–
2	DEC2	ETEC	NAL	256	–	–	+	–	–	–	–
3	DEC3	ETEC	NAL	128	–	–	+	–	–	–	–
4	DEC4	aEPEC	NAL	64	–	–	+	–	–	–	–
5	DEC5	EAEC	NAL	128	–	–	+	–	–	–	–
6	DEC6	aEPEC	NAL,CIP, OFX	> 256	128	32	+	+	+	–	–
7	DEC7	ETEC	NAL,CIP, OFX	> 256	64	64	+	+	+	–	–
8	DEC8	aEPEC	NAL,CIP	> 256	4	–	+	+	–	–	–
9	DEC9	ETEC	NAL,CIP, OFX	> 256	> 256	> 256	+	+	+	+	+
10	DEC10	tEPEC	NAL,CIP, OFX	> 256	64	32	+	+	+	–	–
11	DEC11	ETEC	NAL,CIP, OFX	> 256	32	32	+	+	+	–	–
12	DEC12	EAEC	NAL, OFX	128	–	4	+	+	–	–	–
13	DEC13	ETEC	NAL,CIP	64	16	–	+	+	–	–	–
14	DEC14	aEPEC	NAL,CIP, OFX	> 256	128	32	+	+	+	–	–
15	DEC15	tEPEC	NAL,CIP, OFX	> 256	32	32	+	+	+	–	–
16	DEC16	tEPEC	NAL,CIP, OFX	> 256	128	128	+	+	+	–	–
17	DEC17	ETEC	NAL,CIP	> 256	> 256	–	+	+	–	–	–
18	DEC18	aEPEC	NAL,OFX	> 256	–	> 256	+	+	–	–	–
19	DEC19	ETEC	NAL,CIP	> 256	128	–	+	+	–	–	–
20	DEC20	ETEC	NAL,CIP	> 256	64	–	+	+	–	–	–

All the 5 quinolone (nalidixic acid) resistant strains had a single mutation in *gyrA* at amino acid position 83 (replacement of serine with leucine). All the 15 FLQ resistant strains had double mutations at amino acid position 83 (replacement of serine with leucine) and D87N (replacement of aspartic acid with asparagine) (Table 5). Out of 15 FLQ resistant strains tested, eight resistant strains had a single mutation in *parC* at amino acid position S80I (replacement of serine with isoleucine). No mutation was detected in nucleotide sequences of *gyrB* and *parC* region. Only one isolate harbored both *qnrB* and *aac6’Ibcr* genes.

## Discussion

Diarrhoea is the third leading cause of childhood mortality in India, and is responsible for 13% of all deaths/year in children under 5 years of age [3]. The clinico-observational study was conducted from September, 2015 to November 2017 to identify the potential bacterial pathogens responsible for childhood diarrhoea (≤5 years), drug resistance pattern, genetic markers of virulence and mutation in chromosomal gene. In the present study, 320 non-repeat stool samples from acute diarrhoeal cases were collected and processed for isolation of any bacterial pathogen. Of the 320 diarrhoea cases tested, 157 were from male children, which was lower than detected from females (163), giving a male to female ratio of 0.9:1, lightly different than reported elsewhere [20].

The age and gender wise distribution pattern in 320 children were studied. Most children belonged to 37–48 months of age (30.6%), followed by 7–24 months (24.37%). Mean age of distribution was (Mean ± SD) 34.99 ± 12.77 months, which is concordant with other studies [21–23]. Saeed et al [20] reported maximum number of diarrhoea cases in 49 to 60 months age group (66%), followed by 37 to 48 months of age group (18%). In contradiction to earlier reports [13], the current study reports the majority of diarrhoea cases (72.5%) as well as positive samples (95%) in the age group older than 2 years, particularly those in the age group of 3–5 years as noted above.

The present study, demonstrates a proportional causality for bacterial diarrhoea of 25.6% among under five children in these region among children suffering from gastroenteritis, which is similar to study in other geographical region [24]. Although the morbidity due to bacterial enteric pathogens detected in the current study was low but it was similar to other reports from elsewhere [7, 13, 15, 24]. Moreover, the facility where the study was carried out was a tertiary care hospital and catering services to referred cases from primary and secondary healthcare facilities which might have resulted in low bacterial isolation rate. The current study revealed, majority of acute diarrhoeal cases were associated with common signs and symptoms i.e. fever (52.7%) followed by abdominal pain (51.4%), vomiting (43.4%), dehydration (15.6%) and anemia (9%) similar to study by Raghavan et al [20] who reported fever in 57.9% followed by vomiting in 56.8% cases.

Our current study in accordance with previous other studies [20, 21, 23] revealed, amongst pathogenic isolates, diarrhoeagenic *Escherichia coli* (DEC) was the most common isolate, 77 (24%), followed by *Shigella flexneri* (0.6%), *Vibrio cholerae* (0.6%) and *Salmonella paratyphi B* (0.3%).

Majority of studies showed that *E.coli* as the most important etiological agents of childhood diarrhoea and represents a major public health problem in developing [20]. Among the hospitalized diarrhoeal children up to 5 years of age, DEC was high next to rotavirus and in patients more than 5 years; DEC-mediated diarrhoeal infection was positioned next to cholera [25]. Prevalence of DEC in this study was almost 24% which is higher than the other reports from developing countries and other parts of India [13, 26–29] suggesting variation in distribution of DEC based on geographical location. Among DEC, Enterotoxigenic *E.coli* was found to be 40 (51.9%), Enteropathogenic *E.coli* in 33 (42.8%) and Enteroagrregative *E.coli* in 4 (5.1%). Although EPEC, has been described as the most frequent DEC pathotype in many developing countries [13], our study revealed ETEC as the most prevalent DEC isolated followed by EPEC and EAEC which highlights the need for continued surveillance to understand in depth the distribution pattern. The diarrhoea produced by ETEC is of the secretory type: the disease begins with a sudden onset of watery stool (without blood or inflammatory cells) and often vomiting, which lead to dehydration from the loss of fluids and electrolytes [13, 30].

As observed previously [26], the present study also revealed that the infections caused by *elt* harbouring ETEC strains was less compared to *est* harbouring ETEC. In accordance to earlier studies [26, 31] aEPEC was found to be more than tEPEC across all age group.

In the present study, in comparison to other studies [15] the prevalence of other bacterial pathogens like *Shigella sp., Salmonella enterica* and *V.cholerae* O1 were relatively lower. However, all these bacterial pathogens isolated belonging to these species were multi-drug resistant.

Most of the bacterial pathogen isolated were multidrug resistant which include resistance to ampicillin (85.3%) followed by ciprofloxacin (70.7%), ceftriaxone (60.9%), ofloxacin (58.5%), cotrimoxazole (57.3%) and levofloxacin (56.1%). The rate of resistance among Diarrhoegenic *E.coli* to 1st line, 2nd line and 3rd line of therapeutic drugs were equally high. Earlier studies carried out in the different parts of the world also showed the emergence of MDR among enteric pathogens [12, 21, 23, 32]. Majority of the isolates were sensitive to amikacin, imipenem and tetracycline. In the current study the *Salmonella* isolate exhibited resistance towards nalidixic acid but sensitive to ciprofloxacin. However, in the past decade there have been reports of treatment failures using fluoroquinolones in patients with typhoid fever and the isolate being susceptible to fluoroquinolones and resistant to nalidixic acid in vitro [33]. Studies have reported that plasmid-mediated resistance showed reduced susceptibility to ciprofloxacin (MIC of 0.125–1.0 μg/ml), which could not be picked up by the nalidixic acid test [34]. However, the mechanism is not well understood.

In our study, 62.3% of DEC isolates were ESBL producers which is similar earlier report [35]. The ESBL producing strains showed complete resistance to ampicillin and cephalosporins. This kind of antibiotic resistance pattern might be because of early institution of antimicrobial therapy leading to prevalence of resistant strains [36]. Polymerase chain reaction (PCR) was used to detect *CTX-M3* gene, known as one of the factor for ESBL production. In present study, only 3 (6.1%) isolates were positive for *blaCTX-M3 gene*, which is less in comparison to earlier reports [32, 37] suggesting possible role of other β lactamase genes resistance development, which needs to be studied further**.** During the study period, all the 82 bacterial isolates were resistant nalidixic acid resistant, 59 (71.9%) strains were ciprofloxacin resistant and 48 (58.5%) were ofloxacin resistant. Minimum inhibitory concentration of the isolates ranged between 64 to > 256 μg/mL for nalidixic acid, 4 to > 256 μg/mL for ciprofloxacin and 16 to > 256 μg/mL for ofloxacin.

A subset of DEC isolates resistant to quinolones and fluoroquinolones were subjected to screening for QRDR mutation. Mutations were detected in the gyrA and parC region of the QRDR. No mutation was detected in nucleotide sequences of gyrB and parE region. Only one isolate harboured both *qnrB* and *aac(6′)-Ib-cr* genes. This isolate showed high values of MIC for ciprofloxacin and ofloxacin. *Aac(6′)-Ib-cr* gene, a variant of the common aminoglycoside acetyltransferase is capable of acetylating the piperazinyl substituent of some fluoroquinolones [38], thereby reducing their activities. Similar to earlier reports [32, 39], the present study also detected that quinolone and fluoroquinolone resistance is linked mainly to mutations located in the QRDRs of DNA gyrase (GyrA and GyrB) and topoisomerase IV (ParC and ParE). Current study also supports the previous report [17] of PMQR being a stepwise phenomenon following the multiple mutations in QRDR region.

Carbapenems are recommended as the first option to treat ESBL-resistant strains, however the present study detected emergence of carbapenem resistance among the DEC (2.4%) strains which calls for continuous monitoring to decide the empirical treatment strategy for these common pathogens. Carbapenem resistance in the Enterobacteriaceae is mainly attributed to the production of carbapenemases and the mot predominant being the *bla*_*NDM-1*_. The present report to the best of our knowledge is the 1st report of presence of *NDM-1* in any pathogen from the state of Odisha.

## Conclusion

Knowledge on the etiology is important for effective management and control of diarrhoea. The current study, to the best of our knowledge is first of its kind which demonstrated the etiology of bacterial diarrhoea in children and molecular mechanism of resistance development in Odisha. The present study shows that diarrheogenic *E. coli* are the predominant enteropathogen causing diarrhoea in children less than five years old with majority of the isolates being multi-drug resistance. Emergence of ESBL and carbapenem resistance was also observed among the DEC strains. The data calls for a continuous surveillance system to identify the etiological agents causing diarrhoea which will be instrumental in identifying emerging antimicrobial resistance and for developing treatment guidelines and intervention strategies appropriate for the community.

## Data Availability

The datasets used and/or analyzed during the current study are available from the corresponding author on reasonable request.

## Abbreviations

ADD: Acute diarrhoeal disease
aEPEC: Atypical EPEC
DEC: Diarrhoegenic E.coli
EAEC: Enteroaggregative E.coli
EHEC: Enterohemorrhagic E.coli
EPEC: Enteropathogenic E.coli
ESBL: Extended spectrum β-lactamase
ETEC: Entero toxigenic E.coli
IMNCI: Immunization and Integrated Management of Childhood illnesses
NDM-1: New Delhi metallo β lactamase-1
PMQR: Plasmid mediated quinolone resistance
QRDR: Quinolone resistance determining regions
tEPEC: typical EPEC
